# Adolescent autoimmune encephalitis with dual positive anti-Drebrin and anti-mGluR2 antibodies: a case report

**DOI:** 10.3389/fimmu.2026.1711467

**Published:** 2026-01-21

**Authors:** Anran Liu, Jiarui Huang, Jianqiang Zhang, Junqiang Yan

**Affiliations:** 1 Key Laboratory of Molecular Neurobiology, The First Affiliated Hospital of He Nan University of Science and Technology, Luoyang, China; 2 Medical Intensive Care Unit (MICU), Department of Internal Medicine, The First Affiliated Hospital of He Nan University of Science and Technology, Luoyang, China; 3 School of Clinical Medicine, Henan University of Science and Technology, Luoyang, China

**Keywords:** autoimmune encephalitis, Case report, doubleantibodies, Drebrin antibody, mGluR2 antibody

## Abstract

To date, no research has reported the coexistence of anti-Drebrin antibody and anti-mGluR2 antibody in patients with autoimmune encephalitis (AE). This study presents the first detailed case of AE with dual positivity for these antibodies, aiming to enrich the database of double antibody-positive AE and provide clinical insights for diagnosis and treatment. We report a 15-year-old male patient whose main clinical manifestations were mental abnormalities and epileptic seizures, accompanied by frequent irritability and rambling speech. Serological and cerebrospinal fluid (CSF) analyses showed positive results for Drebrin and mGluR2 antibodies. The titer of anti-Drebrin antibody was 1:1000 (serum) and 1:1 (CSF), while the titer of anti-mGluR2 antibody was 1:32+ (serum) and 1:1+ (CSF). Based on the clinical manifestations and diagnostic results, the patient was diagnosed with AE, with concurrent positive anti-Drebrin and anti-mGluR2 antibodies in both serum and CSF. Significant improvement was achieved following immunotherapies, including steroid pulse therapy and intravenous immunoglobulin infusion. Readmission one month later for relapse responded well to renewed immunotherapy, and the patient was discharged with marked improvement. No recurrence was observed during follow-up.

## 1 Introduction

Autoimmune encephalitis (AE) comprises a group of non-infectious immune-mediated inflammatory disorders of the brain parenchyma often involving the cortical or deep grey matter with or without involvement of the white matter, meninges or the spinal cord (1). In recent years, with the continuous advancement of antibody detection technologies, there has been a gradual increase in reports on double antibody-positive AE. In this study, we present the first case of overlapping autoimmune syndrome characterized by the presence of both Drebrin antibody and metabotropic glutamate receptor 2(mGluR2) antibody. By reporting and analyzing this case, we aim to provide references for clinicians in identifying and managing such diseases, while enriching the published case database of double antibody-positive autoimmune encephalitis and laying a foundation for subsequent large-sample studies.

## 2 Case report

A 15-year-old male patient was admitted to the hospital on August 7, 2024(the day of admission), due to ‘repeated mental abnormalities for 8 days, sudden limb stiffness accompanied by loss of consciousness for 3 hours’. He weighs 51 kg, has no formal education, and no significant past medical, surgical, or drug allergy history. There was also no family history of psychiatric, neurological, autoimmune, or hereditary disorders.

On July 30, 2024 (8 days prior to admission), the patient developed abnormal psychiatric behaviors without obvious predisposing factors, with specific manifestations including persistent irritability, resistance to family members’ instructions, occasional crying episodes, and incoherent, illogical speech—nevertheless, the patient could answer simple questions, recognize family members, and complained of dizziness without medical consultation was sought at this stage. 3 days prior to admission(August 4, 2024), the patient’s psychiatric symptoms worsened, manifesting as sleeplessness at night and repeatedly getting out of bed, prompting the family to bring him to a local psychiatric hospital, where sedation led to the resolution of his psychiatric symptoms that same evening. Concurrently, an electroencephalogram (EEG) performed while the patient was under sedation indicated a moderately abnormal electroencephalogram (**Supplementary Figure**). On admission (August 7), the patient suddenly developed limb rigidity, which was characterized by flexion of both upper limbs and extension of both lower limbs. This rigidity was accompanied by loss of consciousness, which manifested as unresponsiveness to verbal stimuli and sluggish pupillary light reflexes. Bilateral limb convulsions also occurred at the same time. The episode resolved spontaneously after approximately 5 minutes, prompting the family to immediately take the patient to the emergency department of our hospital. On admission, the patient was stuporous (Glasgow Coma Scale [GCS] score: 8; E2V1M5) and uncooperative with the physical examination. Neurological examination revealed equally sized pupils (2.5 mm) with sluggish light reflexes, diffusely increased muscle tone, and a suspected positive pathological reflex in the left lower limb, with no neck stiffness. Cognitive assessment was limited by altered consciousness: the Montreal Cognitive Assessment (MoCA) score for visuospatial/executive function was 0, and other domains were non-assessable.

**Figure 1:**
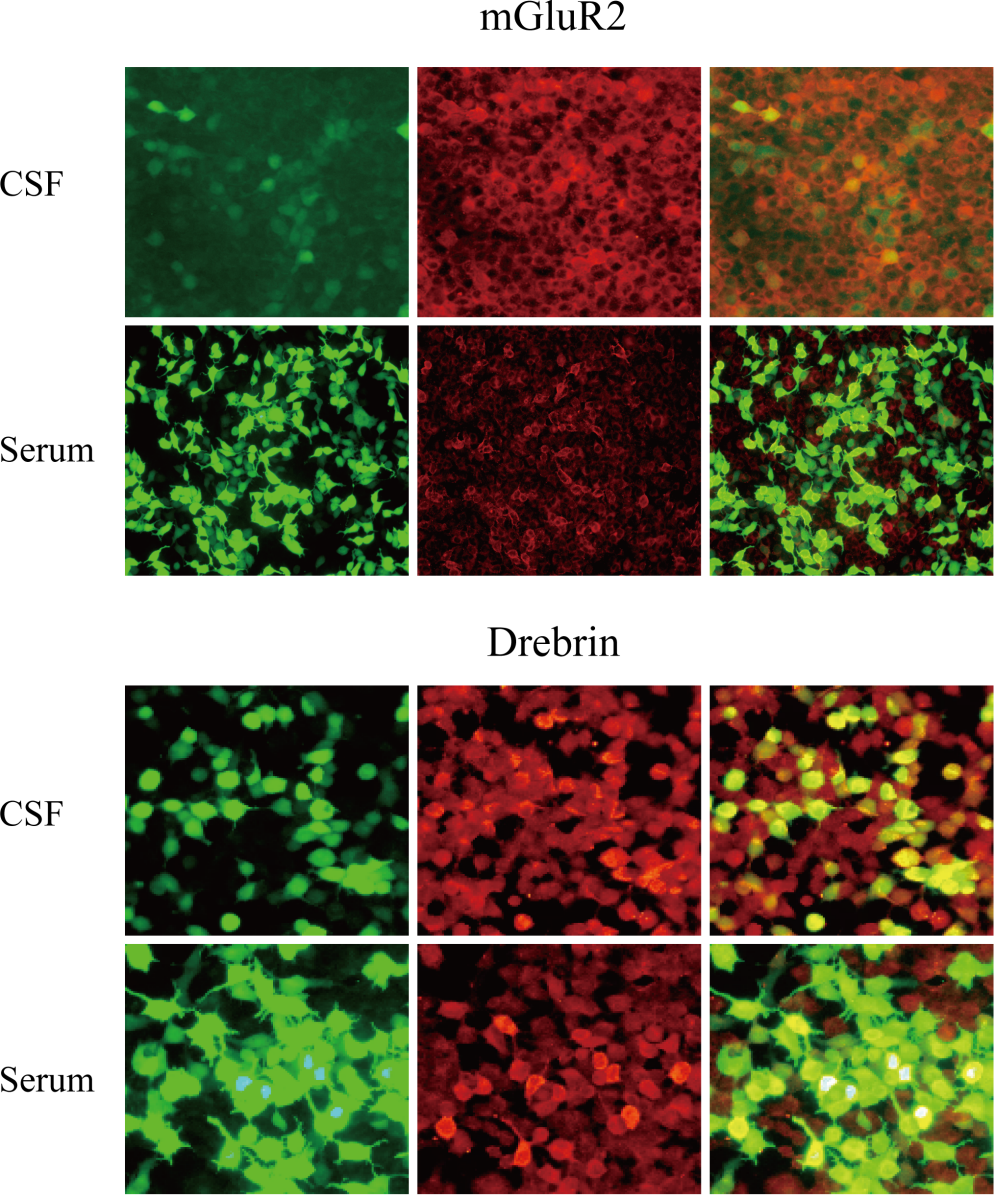
Cell-based assay (CBA) results for serum antibodies. Immunofluorescence images showing that the binding of serum anti-mGluR2, Drebrin antibodies in patients with autoimmune encephalitis. Green: transfected cells (plasmid containing mEGFP). Red: autoantibodies. Original magnification, 200x.

**Figure 2:**
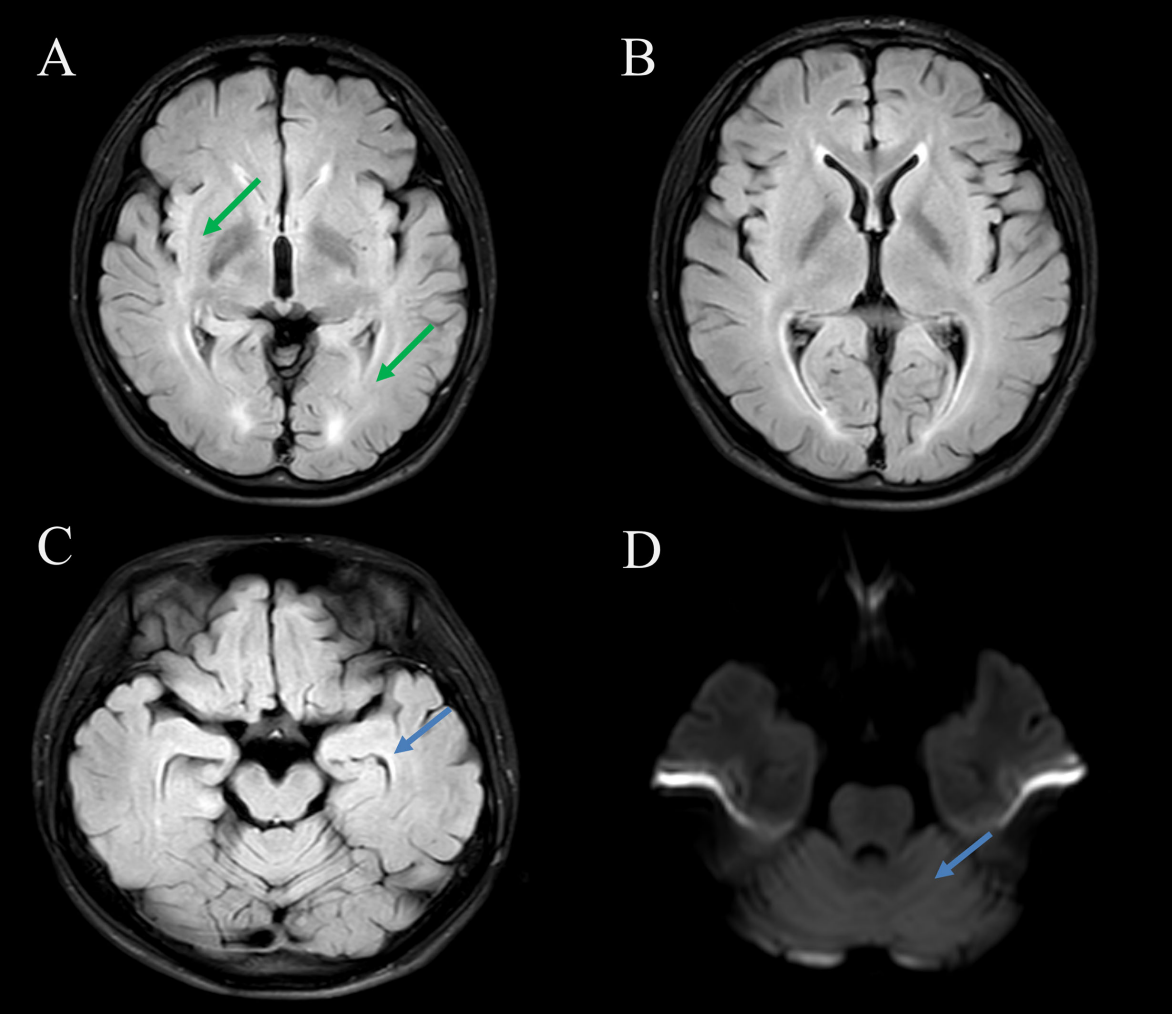
Brain magnetic resonance imaging findings at admission. Magnetic resonance image of the patient’s: **(A, B)** T2/FLAIR sequences showing subtle hyperintensities in the bilateral insula and periventricular regions (indicated by green arrows). **(C)** T2-FLAIR sequence reveals no significant involvement of the limbic system, including the hippocampi (indicated by blue arrows). **(D)** Diffusion-weighted imaging (DWI) shows no apparent abnormalities in the cerebellar regions (indicated by blue arrows).

Emergency cranial computed tomography (CT) revealed a low-density lesion in the right temporal lobe suspected to represent a choroidal fissure cyst. The lumbar puncture showed that the cerebrospinal fluid (CSF)(day 1) was colorless and transparent, with an opening pressure of 140 mmH_2_O (normal range: 80–180 mmH_2_O). CSF routine examination showed a colorless and transparent appearance, a white blood cell count (WBC) of 0×10^9^/L (no white blood cells detected), and a red blood cell count (RBC) of 0.001 × 10¹²/L. CSF biochemical examination indicated that the CSF protein was within the normal range (445.92mg/L). Acid-fast bacillus smear test showed no acid-fast positive bacteria; India ink staining revealed no Cryptococcus neoformans. All antibody tests were performed at Zhengzhou Di’an Medical Laboratory using a commercial cell-based assay (CBA, Eurolmmun) with HEK293 cells transfected with human drebrin or mGluR2 antigens. Positive results were determined by specific immunofluorescence patterns on the cell substrate. Serum samples were diluted at 1:100, and CSF samples were diluted at 1:1. Incubate at 37 °C for 1 hour, then incubate at room temperature in the dark for 30 minutes using Alexa Fluor 488 labeled goat anti human IgG secondary antibody (1:500, Jackson ImmunoResearch). Antibody titers were determined by serial dilution until no fluorescence was detectable. The intra-assay coefficient of variation (CV) was <5%, and the inter-assay CV was <8%. Both anti-mGluR2 antibody (serum titer: 1:32; CSF titer: 1:1) and anti-Drebrin antibody (serum titer: 1:1000; CSF titer: 1:1) were detected (**Figure 1**). On day 9, the patient’s condition stabilized, and he was transferred out of the ICU to undergo a 3.0T cranial MRI (non-contrast scan + DWI). Brain MRI revealed subtle T2/FLAIR hyperintensities in the bilateral insular and periventricular regions (**Figure 2A, B**). **Figure 2C** (including the hippocampus in the limbic system, T2/FLAIR sequence) and **Figure 2D** (cerebellar region, DWI) show no obvious abnormal signals, indicating no significant imaging manifestations of marginal encephalitis and cerebellar inflammation.

Other imaging findings are as follows. Ultrasound examinations of the heart, lower extremity vessels, and abdominal organs, including the liver, gallbladder, pancreas, spleen, and kidneys, performed on day 5, and a chest X-ray from day 7 revealed no obvious space-occupying lesions or structural abnormalities. Other laboratory findings are as follows. On day 1, WBC count of 13.56×10^9^/L, neutrophil percentage of 92%, alanine transaminase (ALT) of 41U/L, aspartate transaminase (AST) of 93U/L, serum creatinine (Scr) of 83μmol/L, and blood urea nitrogen (BUN) of 8.2mmol/L—systemic stress response or initial organ involvement could not be ruled out. Hemoglobin (140g/L), platelet count (191×10^9^/L), and procalcitonin (PCT, 0.06ng/mL) remained normal in all subsequent rechecks, and C-reactive protein (CRP) was <6mg/L before day 12. On day 3, WBC count of 8.21×10^9^/L, neutrophil percentage of 84.6%, AST of 53U/L, and BUN of 6.7mmol/L, trending toward normal, while ALT (38 U/L) and Scr (74 μmol/L) remained unremarkable. On day 7(methylprednisolone was increased to 1000 mg/day), liver and kidney function remained normal without steroid-related injury, while a WBC count of 10.28×10^9^/L and neutrophil percentage of 90.3% were noted, and steroid-induced stress response could not be ruled out. Additionally, serum tumor markers tested on day 7—including neuron-specific enolase (NSE), carbohydrate antigen 19-9 (CA19-9), carbohydrate antigen 125 (CA125), and carcinoembryonic antigen (CEA) were all within normal reference ranges. One day before discharge (day 17), the patient still had normal liver and kidney function, but presented with a WBC count of 29.96×10^9^/L, neutrophil percentage of 94.6%, and elevated CRP (67.83mg/L)—potential subclinical infection, steroid effects, or residual encephalitic activity could not be ruled out; the patient was discharged against medical advice due to financial constraints.

The patient was admitted to the Medical Intensive Care Unit (MICU) for monitoring. Initial treatment regimens included intravenous acyclovir, antiepileptic medications (midazolam and dexmedetomidine infusion, later switched to oral sodium valproate and levetiracetam), and methylprednisolone pulse therapy (500 mg/day starting from Day 1). Upon receiving positive antibody results on Day 5, intravenous immunoglobulin (IVIG, 0.4 g/kg) was immediately added. Due to financial constraints, IVIG was administered for only one day, prompting an increase in the methylprednisolone dose to 1000 mg/day. By day 10, the patient’s consciousness had significantly improved, with a GCS score of 15 and a MoCA total score of 22/30, leading to a steroid taper to 500 mg/day. On Day 11, the patient experienced another epileptic seizure characterized by upward eye deviation followed by generalized tonic-clonic movements of the extremities, accompanied by decreased oxygen saturation. The event lasted approximately 2 minutes and self-terminated, with no subsequent seizures reported. On Day 13, psychiatric symptoms (irritability and incoherent speech) relapsed, prompting the reinitiation of combined therapy with IVIG and methylprednisolone. By day 17, as noted previously, the patient developed marked systemic inflammation (WBC 29.96×10^9^/L, CRP 67.83 mg/L), which was later confirmed by chest CT as a pulmonary infection. Owing to financial difficulties, the family requested discharge against medical advice on Day 18. At discharge, his irritability had lessened but persisted intermittently, with an mRS score of 3 and a MoCA score of 25/30 (mild attention deficit).

**Figure 3:**
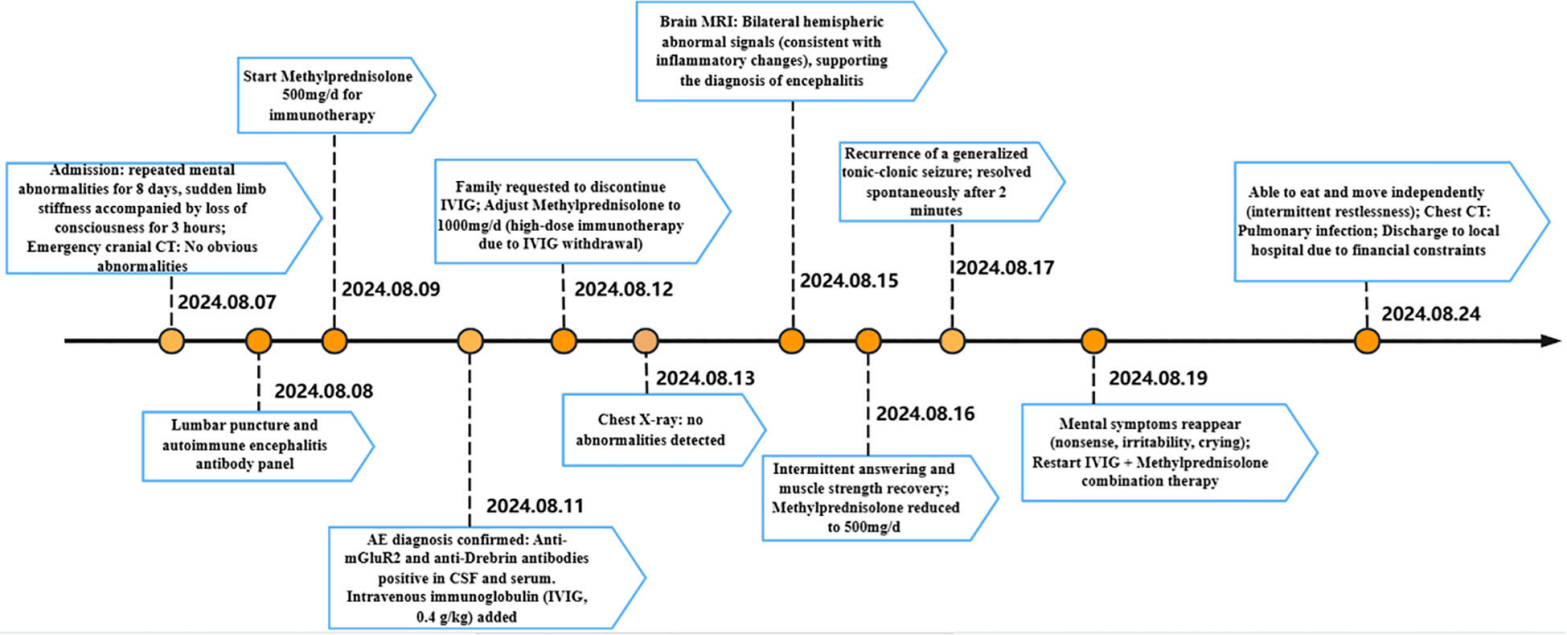
Timeline of clinical symptoms, diagnostic interventions, and treatment. Key events including symptom onset, hospital admissions, diagnostic tests (CT, MRI, lumbar puncture), and treatments (methylprednisolone, IVIG) are shown.

One month after discharge (on September 24), the patient was readmitted to our hospital due to an altered level of consciousness. Repeat autoimmune antibody testing showed that both anti-Drebrin and anti-mGluR2 antibodies were no longer detectable at or above the previous assay threshold. He received further oral corticosteroid therapy, with marked clinical improvement, and was discharged with an mRS score of 1. Favorable prognostic factors, including the patient’s young age, prompt initiation of immunotherapy, robust clinical response, and absence of a detected tumor, contributed to a marked therapeutic response and a favorable prognosis, which was later confirmed during follow-up. The patient’s timeline, along with data on symptom improvement and detailed interventions, as shown in **Figure 3**.

During the most recent follow-up(6 months post-onset), the patient reported normal daily activities with no further epileptic seizures or disturbances of consciousness. His GCS score was 15, MoCA score 28/30, and mRS score 0. Both the patient and his family expressed satisfaction with the outcome but voiced concern about disease recurrence. Follow-up brain MRI demonstrated near-complete resolution of the previous T2/FLAIR hyperintensities in the bilateral insular and periventricular regions (**Supplementary Figure**). Long-term clinical and radiological follow-up is planned for this patient to monitor for potential tumor development.

In summary, this case report presents a 15-year-old male patient with autoimmune encephalitis who presented with acute psychiatric disorders and epileptic seizures. Head MRI showed slightly high signal intensity in bilateral insula and lateral ventricles. Drebrin and mGluR2 antibodies were detected in both serum and cerebrospinal fluid. After immunosuppressive combined treatment, the patient’s neurological and psychiatric symptoms significantly improved. Despite being discharged early due to economic reasons and having elevated non-specific inflammatory markers upon discharge, short-term follow-up showed a good prognosis and no recurrence. This case highlights the importance of considering autoimmune causes in acute neuropsychiatric syndrome. It can also serve as a reminder to clinical imaging not to miss diagnosis during the acute phase due to insignificant images.

## 3 Discussion

To our knowledge, this is the first reported case of AE with positive anti-Drebrin and anti-mGluR2 antibodies in both serum and CSF, accompanied by negative tumor screening results. The patient presented psychiatric abnormalities and epileptic seizures. Laboratory tests detected anti-Drebrin and anti-mGluR2 antibodies in paired samples of serum and CSF. Additional diagnostic tests, including comprehensive tumor screening, yielded negative results. Based on these findings, the patient was finally diagnosed with AE.

Metabotropic glutamate receptors (mGluRs) are a family of G protein-coupled receptors activated by the neurotransmitter glutamate. Based on distinct physiological and pathological characteristics, they are classified into eight subtypes (mGluR1–mGluR8) (2). As a subtype of the mGluR family, metabotropic glutamate receptor 2 (mGluR2) is mainly located on the presynaptic membrane in regions of the central nervous system; its dysfunction can lead to neurological diseases such as epilepsy and Parkinson’s disease (3). Currently, three types of mGluR-associated encephalitis have been identified: anti-mGluR1 encephalitis, anti-mGluR2 encephalitis, and anti-mGluR5 encephalitis. To date, only five previous studies have reported positive cases for the anti-mGluR2 antibody (mGluR2-Ab). Among these, two were case reports describing co-positivity for anti-mGluR2 and other antibodies. One case report described a 61-year-old female who tested positive for both LGI1 and mGluR2 antibodies, presenting with impaired responsiveness, gait disturbance, and speech disorder (4). The other involved an 11-year-old male with concurrent glial fibrillary acidic protein antibody (GFAP-Ab)-associated meningoencephalomyelitis and mGluR2-Ab-associated ataxia (5). The remaining three studies focused on single antibody positivity(involving a total of five female patients): one reported two cases of paraneoplastic cerebellar ataxia in women positive for mGluR2-Ab (6); another described a 56-year-old female with cerebellar ataxia and negative tumor screening results (7); and the third reported two female patients with anti-mGluR2 antibody-associated acute cerebellitis and refractory epileptic seizures (3). A summary of the above cases shows that mGluR2 antibody-mediated autoimmune encephalitis is mainly characterized by clinical manifestations such as ataxia including gait instability and dysarthria. However, in the present study, the patient primarily exhibited bilateral cerebral inflammation, with more prominent disturbance of consciousness and epilepsy than ataxia. Notably, the clinical presentation in this case diverged from the well-documented phenotype of anti-mGluR2 antibody-mediated encephalitis, which is predominantly characterized by cerebellar ataxia, gait instability, and dysarthria (3). Instead, the patient’s most prominent features—including recurrent epileptic seizures, significant neurobehavioral abnormalities (irritability and rambling speech), and bilateral cerebral inflammatory changes on MRI—align more closely with the emerging clinical profile associated with anti-Drebrin antibodies, as previously described in cases of limbic encephalitis and adult-onset focal epilepsy (8).

Drebrin is a cytoplasmic actin-binding protein that functions in neuronal development and is crucial for synaptic function in the brain, as well as for the development, remodeling, and maintenance of dendritic spines (9). Synaptic plasticity is the foundation of advanced brain functions such as learning and memory; Drebrin plays a key role in synaptic plasticity (10). Studies have found that Drebrin expression is decreased in temporal lobe epilepsy (TLE), Alzheimer’s disease, and lung adenocarcinoma (11), while it is increased in glaucoma (12). In patients with TLE, significant hippocampal synaptic reorganization occurs (13). Recently, the University Hospital Bonn reported four cases of patients with a novel autoimmune syndrome, positive for anti-Drebrin antibodies, who presented with adult-onset focal epilepsy, cognitive and behavioral disorders, and limbic encephalitis. They proposed immunosuppressive therapy as a treatment option for suspected encephalitis patients exhibiting neuropsychological impairment, epileptic activity, and positivity for anti-Drebrin antibodies; this approach corresponds to the seizure manifestations and treatment observed in these cases (8). Interestingly, Drebrin regulates group 1 metabotropic glutamate receptor (mGluR)-dependent long-term depression (LTD) in the hippocampus: abnormal expression of Drebrin E promotes group 1 mGluR-dependent LTD, while the developmental switch from Drebrin E to Drebrin A inhibits the excessive activation of mGluR-LTD (14, 15). However, the study only mentioned mGluR1 and mGluR5, and the specific mechanism underlying the interaction between mGluR2 and Drebrin remains to be further explored.

Graus et al. (2016) indicates that the core pathway to diagnose Definite AE is fulfillment of Possible AE criteria plus positive specific anti-neuronal antibodies. First, the case meets the diagnostic criteria for Possible AE as outlined in Graus 2016 Panel 1. It is characterized by an onset of psychiatric abnormalities, and the disease course does not exceed 3 months. Meanwhile, the case presents with newly developed focal CNS signs, seizures not attributable to a known prior seizure disorder, and MRI findings suggestive of encephalitis. Simultaneously, differential diagnostic analyses were conducted to rule out other diagnoses. Primary psychiatric disorders, intracerebral hemorrhage, and space-occupying lesions were excluded via CT and MRI. Initial screening for tumors was performed using tumor markers and ultrasound. Tuberculous and cryptococcal meningoencephalitis were excluded through CSF acid-fast staining and India ink staining. Viral encephalitis was not excluded initially, so acyclovir was administered upon admission; subsequently, based on CSF test results, the treatment was switched to glucocorticoid immunotherapy. Laboratory tests including liver function and renal function assessments were conducted to rule out other metabolism-related diseases. Serological confirmation of dual antibody positivity subsequently served as the pivotal basis for establishing a definitive AE diagnosis (16).

Given the relatively low titer of anti-mGluR2 antibody in this patient and the inconsistency between the clinical manifestations and the typical feature of anti-mGluR2 encephalitis, which is predominantly characterized by cerebellar ataxia, three possibilities need to be considered: asymptomatic anti-mGluR2 result, incidental positivity of anti-mGluR2, or even false positivity. However, the consistent positivity of this antibody in both serum and CSF, along with its seroconversion to negative after treatment, suggests its potential involvement in the immunopathological process. Additionally, considering that cell-based assay (CBA) in **Figure 1** is the standardized method for AE antibody detection, and that the patient could not cooperate with ataxia-related evaluations, thus leaving the possibility of unmanifested clinical features. Nevertheless, given that the titer of anti-Drebrin antibody is significantly higher and its pathogenicity is more well-defined, this suggests that in the context of the coexistence of these two antibodies, the effect of anti-Drebrin antibody may be dominant, or there may be unknown interactions between the antibodies.

It is noteworthy that this pediatric case demonstrated only subtle T2/FLAIR hyperintensities on brain MRI, which aligns with previous literature reporting that a substantial proportion of AE patients may exhibit normal or minimally abnormal MRI findings during the acute phase (17). Specifically, routine brain MRI may be normal or only show subtle non-diagnostic abnormalities in a large subset of AE patients. Although these subtle lesions are non-specific, they are clinically significant when correlated with the patient’s acute psychiatric symptoms, epileptic seizures, and positive autoimmune antibodies, supporting the diagnosis of early inflammatory involvement of the central nervous system (17, 18). This phenomenon underscores the importance of integrating clinical, laboratory, and radiological data for diagnosis, rather than relying solely on MRI findings. Therefore, the absence of significant MRI changes does not preclude the diagnosis of AE, and the decision to administer aggressive immunotherapy is based on the convergence of clinical manifestations and serological findings. Although non-specific, the MRI findings in this case provide valuable clinical reference for recognizing the spectrum of neuroimaging presentations in AE.

Seizures is the common clinical manifestations of AE, especially autoimmune limbic encephalitis, which mainly affects medial temporal lobe structures such as the amygdala and the anterior hippocampus (19). In AE patients experiencing epileptic seizures, antiseizure medications (ASMs) are usually unable to prevent immune-mediated seizures and are generally used for symptom control. However, once the underlying immune-mediated mechanism is correctly identified and treated, these seizures typically resolve (20). These manifestations may be attributed to the synergistic effect of the two antibodies on different brain regions. There is a recognized correlation between the clinical manifestations of AE and antibody types. Moreover, when multiple antibodies act synergistically, the clinical features may become more complex and diverse. Previous studies have also pointed out that in patients with paraneoplastic syndrome, neurological symptoms may precede the diagnosis of tumors by several years. Therefore, even if the initial tumor screening results of AE patients are negative, regular follow-up is still required (4).

During the late hospitalization period (day 17), the patient developed marked systemic inflammation and was diagnosed with a pulmonary infection, raising the issue of interaction between infection and autoimmunity. Against the background of consciousness disturbance caused by AE and immunosuppressive therapy, the secondary pulmonary infection likely exacerbated the patient’s systemic inflammatory state and impaired consciousness, rendering the clinical manifestations more complex. However, the following points support the central role of autoimmune mechanisms in this case. Firstly, the emergence of neurological and psychiatric symptoms such as irritability and cognitive impairment predates the laboratory evidence of this infection. Second, the substantial improvement in the patient’s neurological function was highly correlated in time with the initiation of immunotherapy. Most importantly, the seroconversion of both antibodies in serum and CSF during follow-up was consistent with the response to immunotherapy and could not be explained by the control of infection alone. Therefore, the most plausible interpretation is that this case was primarily autoimmune encephalitis, with a superimposed late infection modifying the severity of its clinical manifestations. This scenario underscores the necessity of maintaining high vigilance and promptly identifying and treating secondary infections in the management of AE.

The near-simultaneous initiation of intravenous methylprednisolone pulse therapy and antiepileptic treatment made it difficult to definitively attribute the clinical improvement to either intervention. We observed that the epileptic seizures resolved within 48 hours of starting antiepileptic drugs, while the psychiatric symptoms and cognitive impairment showed gradual improvement over approximately 10 days of IVIG. This temporal discrepancy suggests that the alleviation of initial symptoms may be directly associated with seizure control by antiepileptic drugs, while immunotherapy may be crucial for long-term inflammation management, recurrence prevention, and promotion of neurological function recovery. Of course, more clinical trials and further research are still required to verify the independent efficacy of immunotherapy.

This case has several limitations. First, the clinical significance of the low-titer anti-mGluR2 antibody remains uncertain, and we cannot completely rule out its role as a concomitant antibody. Second, due to the urgency of the situation, enhanced brain MRI and EEG were not performed, which is a gap in the evidence of this study. Finally, this is a single-case report, and the generalizability of its conclusions needs to be verified by future studies with larger sample sizes. Nevertheless, this case also has notable strengths. To our knowledge, this is the first reported case of autoimmune encephalitis positive for both anti-Drebrin and anti-mGluR2 antibodies worldwide, which expands the antibody and clinical phenotype spectrum of the disease. We have provided extremely detailed clinical courses, imaging, and laboratory data, offering useful references for future similar cases. Meanwhile, it highlights two critical considerations for clinical practice. First, in patients presenting with new-onset seizures accompanied by prominent neurobehavioral symptoms, AE should be included in the differential diagnosis early in the diagnostic workup. Second, an acute neuropsychiatric presentation, which may be misdiagnosed as a primary psychiatric disorder, warrants a thorough neurological evaluation, including cerebrospinal fluid analysis and comprehensive autoantibody testing. These steps are crucial to avoid diagnostic delay and initiate appropriate immunotherapy in a timely manner.

## 4 Conclusion

In summary, we present the first documented case of autoimmune encephalitis seropositive for both anti-Drebrin and anti-mGluR2 antibodies. This report expands the known spectrum of antibody-associated AE and underscores that overlapping autoimmunity can generate distinct clinical phenotypes. When facing acute neuropsychiatric syndromes, clinicians should maintain a high suspicion for AE, pursue comprehensive antibody testing, and prioritize immunotherapy for optimal outcomes.

## Data Availability

The raw data supporting the conclusions of this article will be made available by the authors, without undue reservation.
