# Supplementary material for: Adolescent autoimmune encephalitis with dual positive anti-Drebrin and anti-mGluR2 antibodies: a case report
Source: Front Immunol. 2026 Jan 21;17:1711467. doi: 10.3389/fimmu.2026.1711467 (PMC12867771; doi:10.3389/fimmu.2026.1711467)
Supplement: Supplementary file 1 [file DataSheet1.pdf]

Supplementary Figure

Adolescent autoimmune encephalitis with dual positive anti-Drebrin and anti-mGluR2 antibodies: A case report

List of Supplementary Materials:

Supplementary 1: Pre-admission Electroencephalogram (EEG)；

Supplementary 2: Follow-up Brain Magnetic Resonance Imaging (MRI).

Supplementary 1

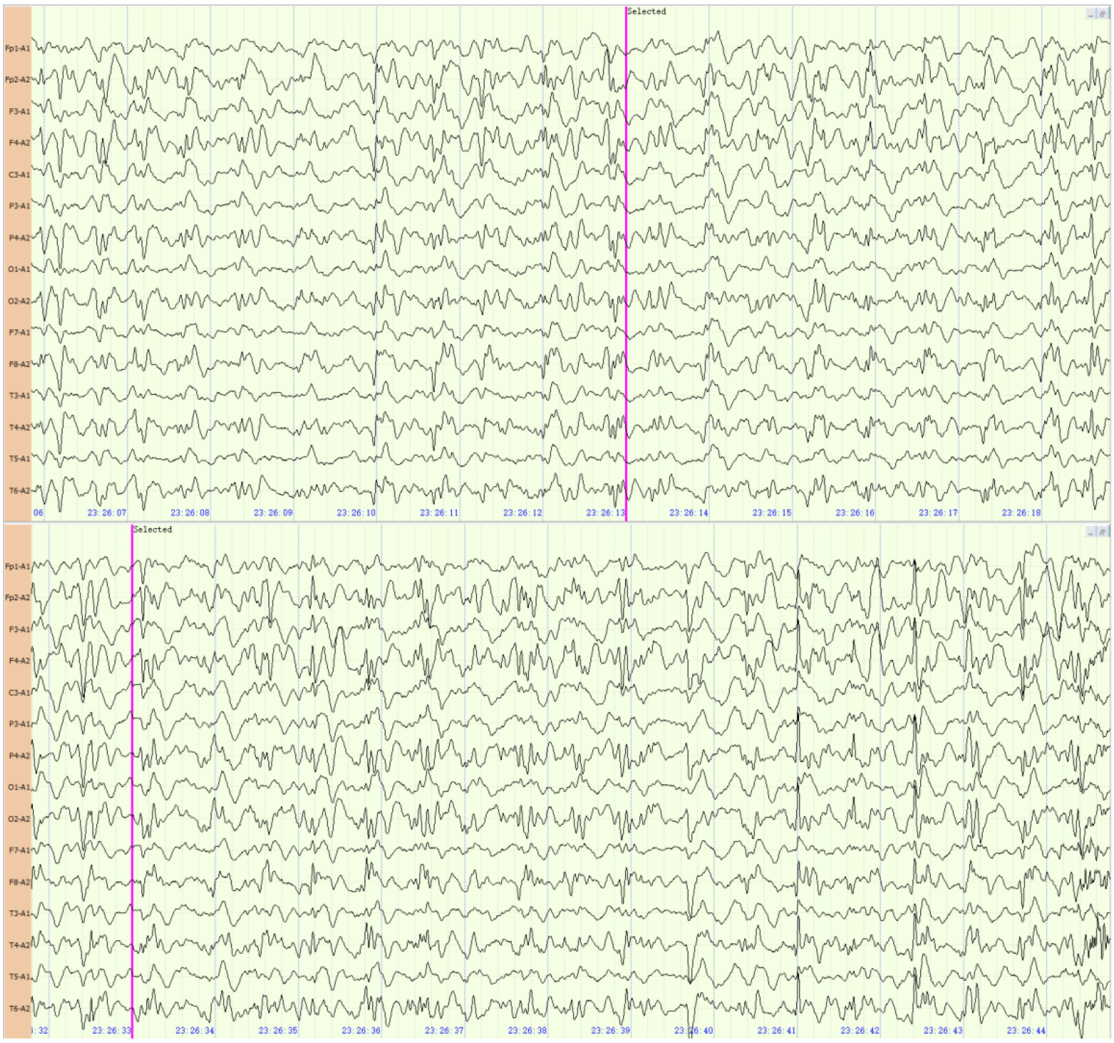

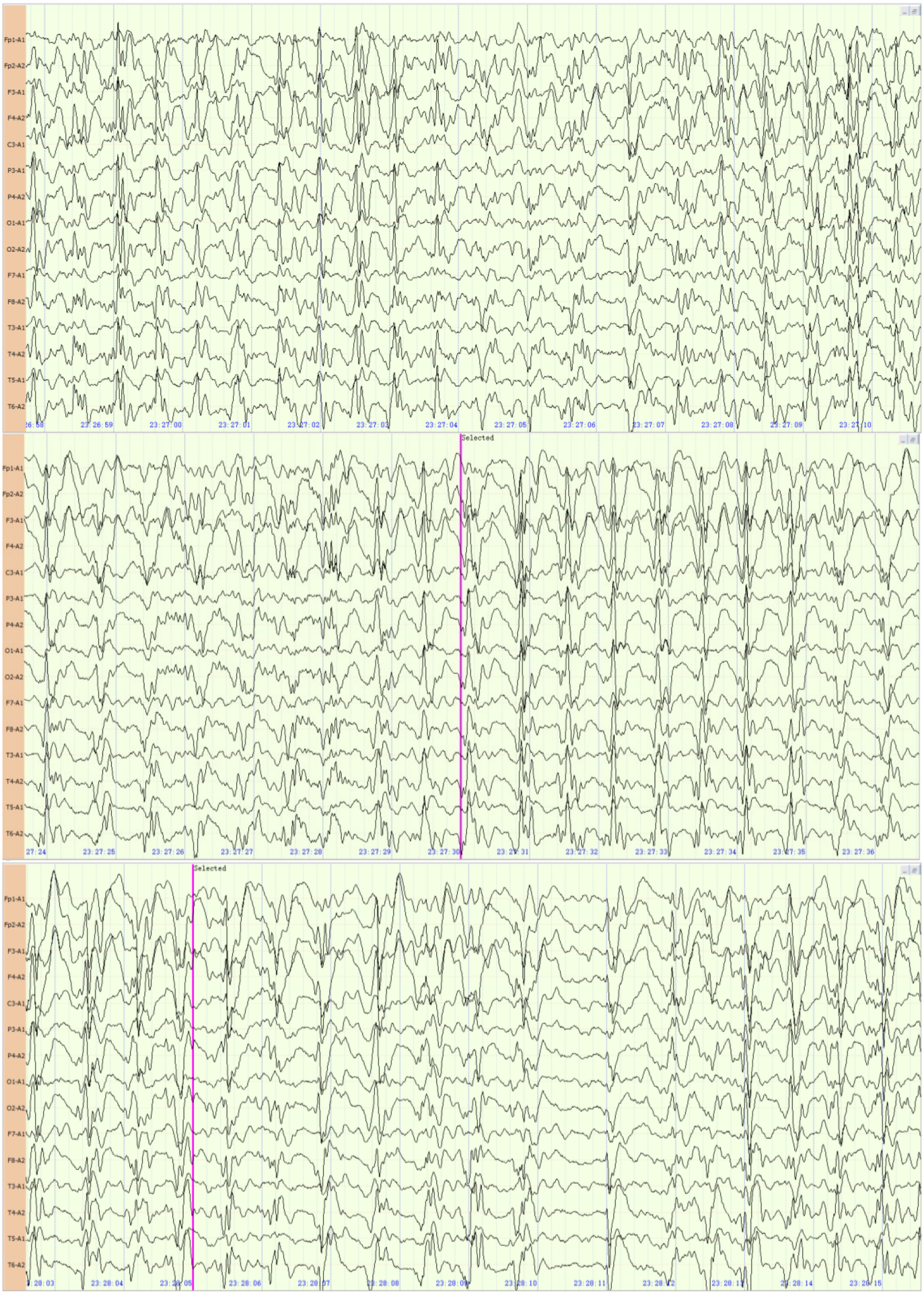
Fp1-A1
Fp2-A2
F3-A1
F4-A2
C3-A1
P3-A1
P4-A2
O1-A1
O2-A2
F7-A1
F8-A2
T3-A1
T4-A2
T5-A1
T6-A2
Selected

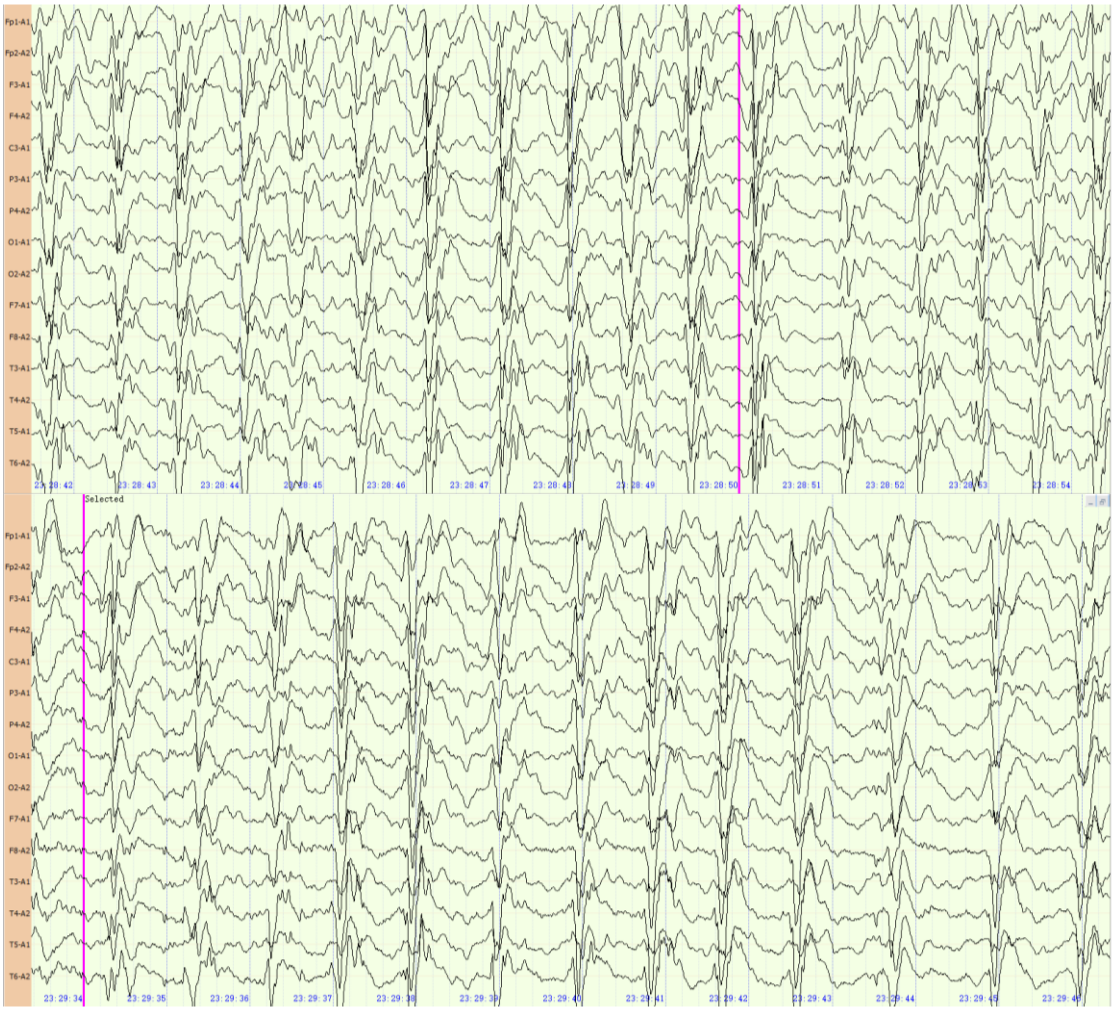

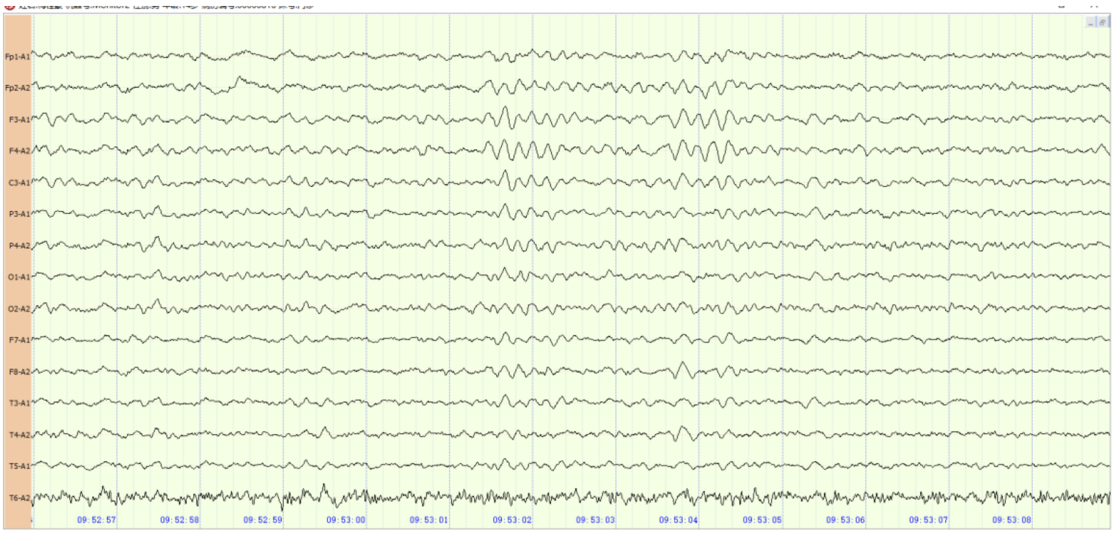

The EEG（under the use of sedatives）：

1. Background EEG Activity: The background EEG was dominated by medium-amplitude (25~60 μV) θ rhythm at 5~7 Hz. A moderate amount of low-amplitude α waves were interspersed.

2. Sleep waves: The sleep stages are obvious, with shallow, medium, and deep sleep stages visible, and low to medium amplitude sleep spindle waves ranging from 12 to 16 Hz can be observed.

3. Pathological waves: Paroxysmal discharges of spikes, sharp waves, spike-slow complexes, and sharp-slow complexes were recorded in all right-sided leads, followed by decreased amplitude in all leads.

Impressions: Moderately abnormal electroencephalogram

Supplementary 2

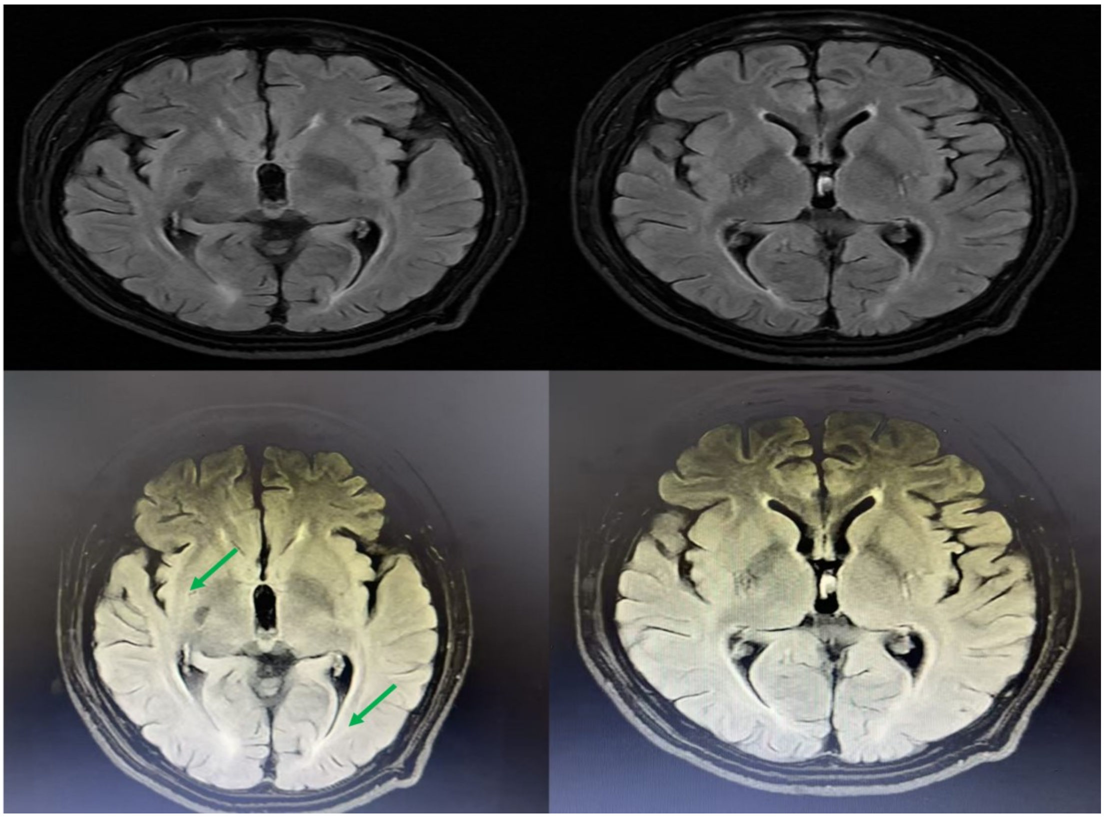

Examination Area and Method: Brain MRI plain scan, 3T:

No significant abnormal MR signals are observed in the brain parenchyma on T2-weighted, and T2-dark-fluid (FLAIR) images and near-complete resolution of the previous T2/FLAIR hyperintensities in the bilateral insular and periventricular regions
